# TRAFFIC-RELATED AIR POLLUTION AND INFECTIONS JOINTLY INCREASE THE RISK OF ALZHEIMER’S DISEASE IN OLDER ADULTS

**DOI:** 10.1093/geroni/igae098.2444

**Published:** 2024-12-31

**Authors:** Svetlana Ukraintseva, Vladimir Popov, Hongzhe Duan, Arseniy Yashkin, Igor Akushevich, Konstantin Arbeev, Anatoliy Yashin

**Affiliations:** Duke University, Durham, North Carolina, United States; Duke University, Durham, North Carolina, United States; Duke University, Durham, North Carolina, United States; Duke University, Durham, North Carolina, United States; Duke University, Durham, North Carolina, United States; Duke University, Durham, North Carolina, United States; Duke University, Durham, North Carolina, United States

## Abstract

Accumulating evidence points to a potentially major role of vulnerability to infections in Alzheimer’s disease (AD) development, although the exact mechanism is not clear. AD is a multifactorial disorder, and the presence of other risk factors may exacerbate the impact of infections on AD. Here we investigated how the interplay between a history of infections and exposure to traffic-related air pollution (TRAP), another risk factor for AD that may influence the brain’s vulnerability to infections, affects the risks of AD and other dementias in UKB participants aged 60-75. Infectious diseases, AD, and other dementia diagnoses were based on ICD9/ICD10 codes. Chronic exposure to TRAP was approximated by closeness (50 meters or less) of participant’s primary residence to a major road. Chi-square, Wilson score interval, Wald interval, Wald risk ratio, and Welch tests, and regression were used to assess statistical significance. We found that the presence of both, a history of infections and exposure to TRAP in UKB participants aged 60-75, was associated with 177% higher risk of AD onset after the age of 75 (risk ratio, RR=2.77, 95% CI [1.86, 4.11]), compared to individuals of the same age who had no history of either of these factors. One mechanism could involve TRAP compromising the integrity of the blood-brain barrier (BBB), potentially increasing the brain’s vulnerability to infections and related damage. This, in turn, might contribute to the promotion of AD pathology.

